# Effects of Choline Chloride, Copper Sulfate and Zinc Oxide on Long-Term Stabilization of Microencapsulated Vitamins in Premixes for Weanling Piglets

**DOI:** 10.3390/ani9121154

**Published:** 2019-12-16

**Authors:** Pan Yang, Huakai Wang, Min Zhu, Yongxi Ma

**Affiliations:** State Key Laboratory of Animal Nutrition, College of Animal Science and Technology, China Agricultural University, Beijing 100193, China; ypan23@163.com (P.Y.);

**Keywords:** choline chloride, copper sulfate, premixes, stability, microencapsulated vitamins, zinc oxide

## Abstract

**Simple Summary:**

Vitamins are essential to animal health and need to be obtained from the diet. As a result of the rapid development of the vitamin industry, microencapsulated vitamins are now available as vitamin sources for premix plants, but vitamins are labile nutrients that are sensitive to the chemical and physical factors that decrease their stability. The current literature on the stability of vitamins in vitamin premixes and vitamin/trace mineral premixes is limited. The most recent recommendations for fortification to overcome losses are provided by a technical bulletin from the company BASF (Badische Anilin-und-Soda-Fabrik). This work has served as the foundation for their recommendations for nearly 20 years; however, the matrix of modern premixes has changed, and conclusions derived from previous studies may no longer hold true, because of changes in vitamin production processing (e.g., microencapsulation) and nutritional content [e.g., choline chloride and high concentrations of copper (Cu) and zinc (Zn) for weanling piglets]. Therefore, the objectives of this study were to determine the rate of vitamin retention in vitamin or vitamin/trace mineral (VTM) premixes, characterize the effects of choline chloride and high concentrations of Cu and Zn on the stability of vitamins and evaluate vitamin stability in vitamin and vitamin/trace mineral premixes during storage.

**Abstract:**

Two in vitro experiments were conducted to investigate the effects of choline chloride, copper sulfate (CuSO_4_) and zinc oxide (ZnO) on the stability of vitamin A (VA), vitamin D_3_ (VD_3_), vitamin E (VE), vitamin K_3_ (VK_3_), vitamin B_1_ (VB_1_), vitamin B_2_ (VB_2_), vitamin B_6_ (VB_6_), niacin, and pantothenic acid in vitamin and vitamin/trace mineral (VTM) premixes for weanling piglets after 0, 1, 2, 3, 6 and 12 months of premix storage. We developed predicted equations to estimate vitamin retention during storage. Two vitamin premixes (with or without choline) were formulated and stored at 25 °C and 60% humidity to establish the storage stability of vitamin premixes. Additionally, four VTM premixes were used to evaluate the effect of choline chloride (0 vs. 40,000 mg/kg) and trace minerals (Low CuSO_4_ + ZnO vs. High CuSO_4_ + ZnO) on vitamin stability in VTM premixes stored at room temperature (22 °C). In general, as storage time increased, residual vitamin activity decreased (*p* < 0.05). The results confirmed that VD_3_, VE, VB_2_, VB_6_, niacin and pantothenic acid were highly stable during storage, while the retention of VA, VK_3_ and VB_1_ was significantly affected by storage time and the presence of choline and high concentrations of Cu and Zn in the premix. After one year of storage, the retention of VE, VB_2_, VB_6_ niacin, and pantothenic acid was more than 90% in vitamin and VTM premixes. The retention of VD_3_ was more than 90% in vitamin premixes and more than 80% in VTM premixes after one year of storage. We conclude that current microencapsulation techniques for vitamin premixes appear to be inadequate to guarantee VA, VK_3_, and VB_1_ concentrations in VTM premixes.

## 1. Introduction

Choline serves as a methyl group donor and a precursor of phosphatidylcholine, which plays an essential role in lipid metabolism in animals [1]. Choline is an essential nutrient for all animals, and a required dietary supplement for some species (e.g., poultry and pig) [1]. Choline chloride is commonly produced by chemical synthesis for use in the feed industry. Copper (Cu) and zinc (Zn) are trace elements that are essential for livestock [2,3]. Traditionally, pig diets have included excessive levels of Cu and Zn to increase the growth rate, feed intake and efficiency of feed utilization in weanling pigs [2,4,5]. Thus, pharmacological levels of Cu (125–250 mg/kg) and Zn (2000–3000 mg/kg) have been frequently added to piglet diets. The most common form of Cu used in feeds to promote growth in pigs is copper sulfate (CuSO_4_·5H_2_O), and the most common form of Zn used in feeds to control diarrhea [2,6] in pigs is zinc oxide (ZnO). In premixes, vitamins are labile nutrients that are sensitive to several factors which affect their stability [7,8,9], and choline chloride, Cu and Zn are stress agents that affect vitamin stability in feeds. However, these factors (choline chloride, high concentrations of Cu and Zn) in premixes have received limited research attention, and it is unclear which vitamins in vitamin or vitamin/trace mineral premixes are vulnerable when choline, CuSO_4_ and ZnO are present. Loss of vitamin activity in vitamin or vitamin-trace mineral premixes during storage may account for hidden depressions in growth, gain efficiency and disease resistance due to subclinical vitamin deficiencies. Microencapsulated vitamins are currently available as vitamin sources, where the chemical structure of these vitamins is protected by microencapsulation, and the nutrients are released upon ingestion [10]. It is believed that microencapsulated vitamins are less reactive and less susceptible to destruction. Therefore, the objectives of this study were to (1) determine the rate of loss of commercial vitamins in vitamin or VTM premixes, (2) characterize the effects of premix storage with choline chloride, CuSO_4_ and ZnO on the stability of vitamins, and (3) evaluated the stability of vitamins in blends and premixes during storage.

## 2. Materials and Methods 

This study was conducted at the State Key Laboratory of Animal Nutrition at China Agricultural University (Beijing, China) and the Ministry of Agriculture and Rural Affairs Feed Efficacy and Safety Evaluation Center (Beijing, China). Approval from the Animal Care and Use Committee was not obtained for this experiment, because no animals were used.

### 2.1. Vitamin Premix Formulation and Treatments

Two vitamin premixes (containing no trace minerals) were formulated with microencapsulated vitamins (purchased from Wellroad Animal Health Co. Ltd., Taiyuan, China). Vitamin premixes were designed to be added as 0.25% of the diet. The vitamin levels were designed to meet or exceed the vitamin requirements of weanling piglets. The manufacturing dates of all vitamins were obtained from the original suppliers to ensure that products were within six months of their manufacture, and not past the recommended expiration. Only one vitamin premix contained 160,000 mg/kg of choline chloride (Table 1). The vitamin concentration in the premixes is based on DSM (company) Vitamin Supplementation Guidelines 2016 and Trouw Nutrition China (2018) Vitamin Recommendations [11,12]. 

### 2.2. Vitamin/Trace Mineral Premix Formulation and Treatments

Four vitamin/trace mineral (VTM) premixes were formulated to contain the same concentrations of vitamins. Microencapsulated vitamin sources were supplied by Wellroad Animal Health Co. Ltd., China. VTM premixes were designed to be added as 1% of the diet. Vitamin levels met or exceeded the Nutrient Requirements of Swine (NRC, 2012) for weanling piglets [13] and were chosen to represent “typical” industry levels according to informal surveys of vitamin levels in commercial premixes from Chinese feed markets. The amounts of vitamins and minerals used in each premix are shown in Table 2. VTM premixes 2 and 4 contained 40,000 mg/kg of choline chloride. VTM premixes 1–4 were formulated to meet or exceed NRC (2012) [13] requirements for copper, iodine, iron, manganese, selenium and zinc for weanling piglets. VTM premixes 3 and 4 contained 20,000 mg/kg of copper (Cu), which was added as CuSO_4_, and 225,000 mg/kg of zinc (Zn), added as ZnO. These levels were chosen because weanling pig premixes have high concentrations of Cu and Zn to improve growth performance and prevent diarrhea [2,5,14]. 

### 2.3. Premix Preparation, Sampling, and Storage

Premixes were manufactured at a commercial vitamin premix plant (Wellroad Animal Health Co. Ltd., Taiyuan, China). The vitamin sources in the present study were retinyl acetate, cholecalciferol, d, l-α-tocopherol acetate, menadione sodium bisulfite (MSB), thiamine mononitrate, riboflavin, pyridoxine hydrochloride, nicotinic acid, d-calcium pantothenate, biotin, folic acid, cyanocobalamin and choline chloride. Each of the two vitamin premixes (vitamin premix with or without choline) was prepared in six separate batches following an identical procedure (7.2 kg per batch). Each batch represented one replicate, and was divided into six thick polyethylene bags weighing 1.2 kg with plastic ties. Each of the four vitamin/trace mineral premixes (VTM premix 1 contained a normal premix formulation for vitamins and trace minerals; VTM premix 2 contained choline chloride and low concentrations of Cu and Zn; VTM premix 3 contained high concentrations of Cu and Zn, but no choline; VTM premix 4 contained choline chloride and high concentrations of Cu and Zn, and was prepared in six separate batches following an identical procedure (18 kg per batch). Each batch represented one replicate and was divided into six thick polyethylene bags weighing 3.0 kg each with plastic ties. Vitamin premixes were stored in a controlled-environment chamber set at 25 °C and 60% humidity. The VTM premixes were stored in a storage room (22 °C). Subsamples from each vitamin and VTM premix were collected from the chamber and storage room at 0, 1, 2, 3, 6 and 12 months. Samples were immediately sent to the Ministry of Agriculture and Rural Affairs Feed Efficacy and Safety Evaluation Center for vitamin analysis. The retention of vitamin A, vitamin D_3_, vitamin E, vitamin K_3_, vitamin B_1_, vitamin B_2_, vitamin B_6_, niacin, and pantothenic acid in vitamin premixes and VTM premixes during storage was determined. Vitamin stability is reported as the residual vitamin activity (% of initial) at each sampling point.

### 2.4. Vitamin Extraction and Assays

The standards used for vitamin assays were retinyl esters, cholecalciferol, α-tocopherol acetate, menadione, thiamine, riboflavin, pyridoxine, niacin and pantothenic acid (Fluka, Sigma–Aldrich, Steinheim, Germany). 

Vitamins A (VA) and E (VE) were determined by the AOAC method [15]. In brief, the sample (2 g) was mixed with papain solution until dispersed, placed in a 37 ± 2 °C water bath, and extracted by methanol. This extract was obtained by HPLC (Agilent 1200 Series; Agilent Technologies Inc., Santa Clara, CA, USA). Vitamin D_3_ (VD_3_) was extracted from samples using the method of 992.26 (AOAC 2012). In brief, a 5 g sample was transferred to a centrifuge tube, to which anhydrous ethanol, ascorbic acid and potassium hydroxide were added. The tubes were placed in a 75 °C water bath. Subsample analysis was carried out by HPLC separation, followed by UV detection at 254 nm. Vitamin K_3_ (VK_3_) in the sample was determined by trichloromethane extraction. The extract was filtered and injected into the HPLC system, and UV detection was performed at a wavelength of 251 nm [16]. Water-soluble vitamins (thiamine, riboflavin, pyridoxine, niacin and pantothenic acid) were extracted from diets using the procedure of Chen et al. with some modification [17]. The sample was weighed to obtain 5 g, extracted with phosphate buffer (PBS), heated in a water bath, and sonicated. The supernatants of the extracted samples were stored at −20 °C until they were tested. These extracted samples were analyzed using a 250 × 4.5 mm, 5 μm, Eclipse Plus C18 column (Agilent Technologies Inc., Santa Clara, CA, USA) on an Agilent liquid chromatograph.

### 2.5. Statistical Analysis

The normality of data was verified using the UNIVARIATE procedure of SAS (SAS Institute, Cary, NC, USA). The BOXPLOT procedure of SAS was used to check for outliers. If outliers were observed, they were examined. If the outliers were not due to errors in measurement or data entry, then they were excluded. If the outliers were due to such errors, then they were corrected. Data were analyzed using the MIXED procedure of SAS. Data from vitamin premix storage were analyzed by *t*-test to compare means. The data from vitamin/trace mineral premixes were analyzed using a completely randomized design with a 2 × 2 factorial arrangement of treatments. The main effects were choline chloride (0 vs. 40,000 mg/kg) and trace minerals (Low CuSO_4_ + ZnO vs. High CuSO_4_ + ZnO). Choline chloride, trace minerals (CuSO_4_ and ZnO), and their interaction served as fixed effects. Only the main effects are discussed for responses without significant interactions. Analysis of variance (ANOVA) was conducted to evaluate the effect of storage time on the retention of vitamins. The LSMEANS statement was used to calculate treatment means, and the means were separated using the Tukey test. Results were considered significant at *p* < 0.05. Excel 2016 (Microsoft Corporation, Redmond, Washington, DC, USA) was used to indicate the general course or tendency of data, and the data were used to develop predicted equations using the PROC REG and PROC NLIN procedures of SAS. The *R*^2^ and the root-mean-square error of prediction (RMSEP) were used to define the best-fit equations.

## 3. Results and Discussion

### 3.1. Vitamin Recovery Method Validation and Storage Stability Test

The methods for analyzing vitamin concentration in the samples were validated for repeatability, between-day precision, long-term precision, limits of quantitation and linearity (data not shown) by the staff of the Ministry of Agriculture and Rural Affairs Feed Efficacy and Safety Evaluation Center (Beijing, China). Initial values (Table 3) were within 10% of their formulated targets, which is consistent with the acceptable analytical variation and recovery of other vitamins previously described by the Association of American Feed Control Officials [18]. When developing the experimental protocol, we took into account the recommended shelf-life of vitamins, and designated five time-points to test vitamin stability. This timing was also convenient for comparison with previous studies, and for developing predicted equations to estimate vitamin retention.

### 3.2. Effects of Storage Time on the Stability of Vitamins in Vitamin and VTM Premixes

There were no significant effects of storage time on VA, VD_3_, VE, VB_6_, niacin and pantothenic acid activities in vitamin premixes without choline chloride or on VD_3_, VE, niacin and pantothenic acid activities in vitamin premixes with choline chloride (Table 4, Table 5, Table 6, Table 7, Table 8 and Table 9). However, storage time was a significant factor that affected the retention of VA activity in vitamin premixes with choline chloride, VK_3_ activity in two vitamin premix formulations (Table 4 and Table 10) and VB_1_ and VB_2_ activities in vitamin premixes with or without choline chloride, as well as in four tested VTM premixes (Table 11 and Table 12).

Zhuge and Klopfenstein conducted experiments on vitamin storage at room temperature, and they reported that 56–57% of VA was destroyed after one month of storage, and 50%–54% of VB_2_ was destroyed after 27 weeks of storage [19]; there are high losses compared with the results of our study. The high losses reported in the previous study may be easily explained by a limitation in production processes three decades ago that affected vitamin stability. In Zhuge and Klopfenstein’s experiment [19], gelatin-coated VA and VB_2_ (containing 50% riboflavin) were used as vitamin resources. Gelatin as a wall material did not sufficiently protect VA, and carriers of VB_2_ (50% purity) were an important factor of its stability. In the present study, the retention of niacin was more than 90% after one year of storage (Table 8), which is consistent with Zhuge and Klopfenstein [19], who reported that the retention rate of niacin during storage was 91–96% during storage. This was not surprising to us, because niacin has been reported to be the most stable of the B vitamins when added to feed or premixes [20], owing to its a stable molecular structure, which reduces its oxidation during storage. Hughebaert [21] determined the stability of different vitamin K formulations in a multi-vitamin during storage. After four months, MSB retained 33% of its original activity. The retention of VK_3_ in the vitamin premix was higher than that reported by Hughebaert [21], but this is consistent with our conclusion that VK_3_ products are unstable in storage. VK_3_ (menadione) is a synthetic quinone derivative of naphthalene, which is unstable. According to BASF data (BASF 1994, cited by Whitehead), extending the storage time reduces the vitamin concentration in VTM premixes. BASF reported that the retention of VA, VD_3_, VE, VK_3_, VB_1_, VB_2_, VB_6_, niacin and pantothenic acid after one month of storage was 85%, 91%, 95%, 64%, 70%, 95%, 92%, 95% and 95%, respectively [22]; after six months of storage, their retention was 58%, 65%, 82%, 0%, 27%, 56%, 56%, 58% and 58%, respectively [22]. In stark contrast, we observed that VD_3_, VE, VB_2_, VB_6_, niacin and pantothenic acid were relatively stable, and losses of VA, VK_3_ and VB_1_ did not exceed 50%. This disparity result is easily understood when one considers that the vitamins in the current study were microencapsulated, which improves vitamin stability in storage. Microencapsulation is a process in which molecules are coated with a continuous film of polymeric material [10]. Substances that are sensitive to oxygen, moisture, or light can be stabilized by microencapsulation, and experiments in recent years have revealed enhancements in the stability of vitamins in stress conditions. The retention of VB_1_ was reported to be 83%–87% in a normal premix after six months when stored at 20 °C [23,24], which is similar to our results. Shurson et al. reported that vitamin activity in vitamin and VTM premixes decreased with prolonged storage time. They observed that the retention of VA, VD_3_, VE, VK_3_, VB_1_, VB_2_, VB_6_ and niacin in vitamin premixes was 86%, 87.92%, 93.56%, 75.96%, 89.44%, 86.68%, 76.52%, and 84.24%, respectively. In the same study, they observed that the retention of VA, VD_3_, VE, VK_3_, VB_1_, VB_2_, VB_6_ and niacin in VTM premixes was 64.12%, 82.08%, 95.52%, 95.36%, 68.4%, 89.04%, 65.44% and 87.04%, respectively [25]. Comparing the vitamin retention reported by Shurson et al. with that in the BASF technical bulletin (BASF 1994, cited by Whitehead) shows that vitamin stability improved between 1994 and 2011. In the study by Shurson et al., the stability of VD_3_, VE, VB_2_, niacin and pantothenic acid was higher than that of other vitamins, which is consistent with our observation [25].

Mooney and Aldrich conducted a shelf life study with vitamin premixes in ambient storage conditions (20 °C and 50% humidity) [26]. They concluded that storage time affected vitamin content, since after six months of storage under ambient conditions, the retention of VA and VB_1_ in the vitamin premix was 76% and 40%, respectively. Storing vitamins for long periods has been considered to negatively affect the vitamin activity in vitamin or VTM premixes. However, the results of our study indicate that the storage time had little influence upon the stability of vitamin premixes. Microencapsulation protected the core vitamins in premix from reactions with the environment, thereby increasing its shelf life. VE, niacin and pantothenic acid, and VB_2_ were more stable than the other vitamins in VTM premixes (Table 6, Table 8, Table 9 and Table 12). These results corroborate Coelho’s finding that common commercial forms of VE, VB_2_, niacin and pantothenic acid were stable to heat and air [27]. In addition, microencapsulation is more restricted to the entrapment of VE, VB_2_, niacin and pantothenic acid to improve the product’s performance and enhance its shelf life. 

### 3.3. Effects of Choline Chloride on the Stability of Vitamins

Choline chloride did not significantly influence the retention of VD_3_, VE, niacin, pantothenic acid, VK_3_, VB_1_, and VB_2_ in vitamin premixes (Table 5, Table 6, Table 8, Table 10, Table 11 and Table 12). During the first six months of the study, the concentrations of VA and VB_6_ in vitamin premixes with and without choline chloride showed no significant difference (Table 4 and Table 7). After one year of storage, it became obvious that VA and VB_6_ were more stable in the vitamin premix without choline chloride than in the sample with choline chloride (*p* < 0.05). For VTM premixes, no negative effects of choline chloride were observed on the retention of VD_3_, VE, VB_6_, niacin, pantothenic acid, VK_3_ and VB_2_, after 12 months of storage (Table 5, Table 6, Table 7, Table 8, Table 9, Table 10, and Table 12). The supplementation of choline chloride significantly reduced the concentrations of VA in VTM premixes after 3, 6 and 12 months of storage (Table 4). After two months, the concentrations of VB_1_ in VTM premixes without choline chloride were lower than they were at the beginning of the study (*p* < 0.05) (Table 11). As the period of storage was extended to 3, 6 and 12 months, the VB_1_ content in VTM premixes (premix containing vitamins, choline chloride and high concentrations of Cu and Zn) gradually decreased (Table 11). 

Choline chloride has been reported to significantly affect vitamin activity. Choline is considered a stress agent that affects vitamins which dissolve easily in water because it is hygroscopic, and can attract moisture to vitamin or VTM premixes. The concentration of choline chloride in feed is usually higher than the micro-ingredient level, and problems related to both physical and chemical properties can be expected when choline is added to vitamin or VTM premixes [27]. A technical bulletin from BASF authored by Coelho (2002) reported that the stability of vitamins in a premix depended on the presence of choline chloride. After six months of storage, the recovery of VA, VD_3_, VE, VK_3_, VB_1_, VB_2_, VB_6_, niacin and pantothenic acid in a vitamin premix without choline chloride was 95%, 96%, 98%, 99%, 99%, 99%, 99%, 99% and 99%, respectively [9]; with choline chloride in the premix, the recovery after six months of storage was 90%, 93%, 98%, 68%, 73%, 85%, 83%, 85% and 86%, respectively [9]. In addition, the negative effects of choline chloride on vitamins in a VTM premix were more significant. After six months of storage, the recovery of VA, VD_3_, VE, VK_3_, VB_1_, VB_2_, VB_6_, niacin and pantothenic acid in a VTM premix without choline chloride was 80%, 84%, 84%, 70%, 80%, 83%, 81%, 83% and 88%, respectively [9]. However, in a VTM premix with choline chloride, the recovery after six months was 70%, 73%, 77%, 50%, 48%, 71%, 68%, 70% and 67%, respectively [9]. In the present study, the retention of VA, VD_3_, VE, VB_1_, VB_2_, VB_6_, niacin and pantothenic acid in the vitamin premix without choline chloride was similar to the results of Coelho, but the retention of water-soluble vitamins (VB_1_, VB_2_, VB_6_, niacin and pantothenic acid) in vitamin premixes with choline chloride was higher than Coelho’s results. Regardless of whether VTM premixes had choline chloride, the retention of the examined vitamins was higher than that reported by Coelho. The reason may be that commercially-available vitamins have good stability. Water-soluble vitamins are easily attacked in premixes containing choline chloride. However, the polymeric material used in the microencapsulation of vitamins resists moisture, which reduces the reactivity and incompatibility of vitamins with their surroundings, and enhances their stability in storage. Actually, the practice of microencapsulation is not a new technology. Microencapsulation is defined as small solid, liquid, or gas particles coated with or entrapped within a continuous film of polymeric material [10,28]. Microencapsulated vitamins are stable in the present study because the wall materials effectively protect the core material from the environment.

The chemical structure of VA used in this study was that of a commercially-available retinyl ester. The esterification of retinol with acetic acid produces retinyl acetate; although it has a protected hydroxy group, it still has five double bonds that are susceptible to oxidation. In addition, choline chloride absorbs or releases water vapor, which softens the coating of VA and provides access to oxygen and other compounds, thus destroying VA by accentuating chemical reactions. In the present study, VA was markedly more stable in vitamin premixes without choline chloride than in vitamin premixes with choline chloride after 12 months of storage, which is consistent with a previous report [29]. However, the results of the present study show that the retention of vitamin activity was approximately 90% in vitamin premixes with or without choline chloride after one year; this retention is higher than the result of Tavčar-Kalcher and Vengušt, who reported VA losses of 61% and 47% in premixes with and without choline [29]. The reason for the high retention of VA in the present study may be that VA microencapsulation improved its stability, which is supported by Gonçalves et al. [10].

Menadione (VK_3_) is unstable. Premix plants do not utilize its pure form, but rather use sodium bisulfite and its derivatives. The most common menadione compound used in the industry is menadione sodium bisulfite (MSB), a water-soluble salt. VK_3_ is very sensitive to moisture and trace minerals, and choline chloride is particularly destructive to VK_3_ [9]. Choline chloride increases the leaching of VK_3_ in premixes, and prolongs oxidation–reduction reactions. In a previous study, the stability of VK_3_ supplements in premixes and diets was impaired by moisture, choline chloride and trace elements. VK_3_ lost almost 80% of its bioactivity if it was stored for three months in a VTM premix containing choline, but losses were far less if stored in a similar premix without choline [30]. Hughebaert studied the stability of VK_3_ in vitamin premixes containing choline chloride and trace minerals at room temperature. After four months, VK_3_ loss almost exceeded 70% [21]. Stability data published by BASF (1994, cited by Whitehead) showed a 64% retention of VK_3_ in premixtures containing choline chloride and trace minerals after one month, and 0% after six months of storage [22]. Tavčar-Kalcher and Vengušt [29] reported that VK_3_ was more stable in vitamin premixes containing no choline chloride than in vitamin premixes with choline chloride, and the loss of VK_3_ was almost 100% in vitamin premixes containing choline after 12 months of storage. Historically, choline has been an important stress factor for VK_3_ storage. We observed that the stability of VK_3_ was poor in all tested vitamin premixes and VTM premixes, and choline and high levels of Cu and Zn did not affect VK_3_ retention (*p* > 0.05) (Table 10). The reason that the VK_3_ was not affected by choline could be that the coated material reduced the opportunity for a chemical reaction between VK_3_ and choline, but the composition of vitamin premixes, the VK_3_ source and the microencapsulation process might also have contributed to the loss of VK_3_ observed in the present study. As mentioned above, microencapsulated VK_3_ is relatively stable in premixes that contain large quantities of choline chloride, but we should consider and pay attention to storage time when vitamin or VTM premixes are stored for an extended period of time.

Thiamine (VB_1_) is an essential vitamin for animals. Frias et al. reported that VB_1_ was stable under acidic conditions, but was destroyed rapidly above pH = 7.0, even at room temperature [31]. The two main supplemental forms of thiamine added to feed are thiamine hydrochloride and thiamine mononitrate. This thiamine mononitrate is used more often in feed because of its higher stability compared with thiamine hydrochloride [9]. The structure of thiamine helps to explain thiamine’s instability in storage. The methylene bridge connecting the pyrimidine and thiazole moieties is easily broken [32], and the thiazole moiety is less stable than the pyrimidine structure and easily cleaved by hydrolysis. The inclusion of choline chloride in a vitamin premix contributes to VB_1_ instability during storage. Stability data published by BASF (1994, cited by Whitehead) showed VB_1_ retention of 70% after one month and 27% after four months of storage in premixes containing choline chloride and trace minerals [22]. VB_1_ is most likely dissolved in water because the hydrogen atoms bond to the hydroxyl groups and amine tertiary structures. Therefore, VB_1_ is susceptible to degradation during storage because of its ability to dissolve in water. Vitamin stability can be improved by excluding choline chloride from a vitamin/trace-mineral premix.

### 3.4. Effects of High Concentrations of Cu and Zn on the Stability of Vitamins

High concentrations of Cu and Zn in VTM premixes had no effect on the retention of VD_3_, VE, VB_6_, niacin, pantothenic acid, VK_3_, and VB_2_, retention (Table 5, Table 6, Table 7, Table 8, Table 9, Table 10 and Table 12). Conversely, supplementation with high concentrations of Cu and Zn significantly reduced the concentrations of VA and VB_1_ in VTM premixes after 2, 3, 6 and 12 months of storage (Table 4 and Table 11).

Post-weaning diarrhea is one of the most common causes of morbidity and mortality in weanling pigs and hence greatly impairs their growth performance [2,4,5]. In commercial conditions, feeding piglets with high concentrations of Zn and Cu stimulates their average daily gain, decreases the feed conversion factor, improves the digestibility of dietary nutrients and growth performance, and decreases the incidence of diarrhea [2,6]. However, Zn and Cu are heavy metals and tend to accumulate in the soil, leading to the serious environmental pollution of soil and tap water. Furthermore, high zinc concentrations (2000–3000 mg/kg feed) in feed may have an impact on the development of antimicrobial resistance. In the current study, high levels of CuSO_4_ (more than 20,000 mg/kg of Cu in premix to promote growth) and ZnO (more than 225,000 mg/kg of Zn in premix to decrease the incidence of diarrhea) in vitamin/trace mineral premixes resulted in the degradation of vitamins. Vitamin stability is reduced in the presence of certain trace minerals [19,25,33]. In the present study, blending vitamins with trace minerals to form vitamin/trace mineral premixes increased the loss of vitamin activity during prolonged storage periods (Table 4, Table 5, Table 6, Table 7, Table 8, Table 9, Table 10, Table 11 and Table 12). Trace minerals produce redox reactions that cause the oxidation of vitamins. Trace minerals vary in their redox potential: copper, iron and zinc are the most reactive, and selenium, iodine and manganese are less reactive minerals [9]. Reactive trace minerals reduce vitamin activity by oxidizing the vitamins. First, the metallic-like nature of trace minerals reduces the crystals of vitamins to smaller particles by eroding their protective coating. The smaller particles provide increased the surface areas of vitamins for reactions between vitamin particles and trace mineral particles. Dove and Ewan [34] also reported that a high concentration of Cu (250 mg/kg feed) or Zn (1000 mg/kg feed) increased vitamin loss. Lu et al. reported that a high concentration of Cu sulfate promoted the undesirable oxidation of VE in feeds [35]. In the production of commercially-available VE, the hydroxy group of VE is protected by the formation of an ester, as in α-tocopherol acetate. The obtained tocopherol acetate is resistant to oxygen, since it lacks double bonds and free hydroxy groups [36]. In addition, Dove and Ewan [34] found that α-tocopherol acetate was relatively stable in pig diets, but 250 ppm of Cu in the diets decreased the stability of α-tocopherol acetate. Intriguingly, we did not find significant decreases in VE content in the four VTM premixes, likely because of the microencapsulation technology used to protect VE. Microencapsulation can reduce the reactivity and incompatibility of compounds with the exterior, thus enhancing their stability in conditions involving heat, light, moisture and oxygen, among other stressors, and decreasing the loss of vitamins during storage.

Coelho [9] reported that the stability of coated VK_3_ in premixes was influenced by the presence of trace minerals. Our data indicate that the stability of microencapsulated VK_3_ in vitamin or VTM premixes was not affected by choline and high concentrations of Cu and Zn. The reason may be that the microencapsulation process of VK_3_ provides a better defense against potential damage. Loss of VB_6_ was relatively low when stored in vitamin premixes, but in the presence of trace minerals, VB_6_ rapidly degraded because the stability of VB_6_ is highly affected by the presence of trace minerals [1]. VB_6_ is normally stable to atmospheric oxygen and heat, but it decomposes rapidly in the presence of metal ions. In addition, VB_6_ is sensitive to light, particularly in neutral and alkaline solutions [9]. In VTM premixes, VB_6_ can lose bioactivity, particularly when minerals in the form of carbonates or oxides are present [20]. Further, Shurson et al. reported higher losses of activity in vitamin premixes blended with inorganic trace minerals, compared with a vitamin premix without inorganic trace minerals stored for 120 days [25]. In a vitamin/inorganic trace mineral premix, they observed 8.97%, 10.16%, 7.90% and 8.64% loss of activity per month for VA, VK_3_, VB_1_, and VB_6_, respectively [25]. In a vitamin premix, they observed 3.50%, 6.01%, 2.64% and 5.87% loss of activity per month for VA, VK_3_, VB_1_ and VB_6_, respectively [25]. In contrast, we observed that microencapsulation was effective in improving vitamin stability in the presence of high concentrations of Cu and Zn compared with previous studies. For the microencapsulation of vitamins, one of the favorable factors is the use of a lipid matrix. because it can prolong the viability of vitamins during storage by blocking their exposure to stressors (choline or high concentrations of Cu and Zn).

### 3.5. Prediction Equations for Vitamin Retention in Premixes during Storage

Prediction equations have been widely used to estimate values through regression analysis and deep learning. The use of such equations potentially decreases the requirement for time-consuming, expensive experiments and improves precision when estimating values. Regression analysis was used to establish prediction equations for vitamin content in premixes on the basis of storage time. The *R*^2^ and RMSEP were used to compare the prediction accuracy, and the equations with the greatest *R*^2^ and the smallest RMSEP were chosen as the best-fit model [37]. The equations and their associated *R*^2^ values and RMSEP are presented in Table 13. For VTM premix 1 (normal premix formulation for vitamins and trace minerals), the equations for predicting the retention of VA, VD_3_, VK_3_, VB_2_, VB_6_, niacin and pantothenic acid explained over 95% of the variation in vitamin retention. Equations for predicting the retention of VD_3_, VK_3_, VB_6_ and niacin in VTM premix 2 (premix containing choline chloride and low concentrations of Cu and Zn) had high *R*^2^ values compared with the equations for VA, VE, VB_1_, VB_2_, and pantothenic acid. For VTM premix 3 (premix containing high concentrations of Cu and Zn but no choline), the equations for predicting the retention of VD_3_, VK_3_, VB_1_, VB_6_ and pantothenic acid had high *R*^2^ values and a better fit than VA, VB_2_, VE and niacin. For VTM premix 4 (premix containing choline chloride and high concentrations of Cu and Zn), the equations had higher *R*^2^ values for the predicting retention of VD_3_, VK_3_, VB_2_, VB_6_ and niacin than those for predicting the retention of VA, VE, VB_1_ and pantothenic acid during storage. Obviously, the degradation of vitamins over a month did not follow a linear trend. Time has been shown to be an important prediction estimator of vitamin retention during storage [27]. Thus, time was considered to be the best predictor in the present study. In addition, we used SAS procedures (SAS Institute, Cary, NC, US) to test for the convergence criterion and fit the curve with the exponential function model. This result is consistent with Giannakourou et al. who reported an exponential fitting for the measurement of vitamin loss, and their developed models were validated with fluctuating time [38]. On the other hand, the shelf life of a product is determined by the stability of its most unstable ingredient [11,12]. Our results indicate that VA, VK_3_ and VB_1_ are the ingredients that limit the shelf life of VTM premixes. To our knowledge, the present study is the first to establish prediction equations for the retention of various vitamins in vitamin and VTM premixes during storage. Therefore, the equation obtained in this study is easier to apply than the tabular values reported in previous studies for predicting vitamin retention. 

## 4. Conclusions

The majority of the examined vitamins were more stable than VA, VK_3_ and VB_1_ in vitamin premixes, and VK_3_ was very unstable. Choline chloride and high concentrations of Cu and Zn significantly reduced the stability of VA and VB_1_. The current microencapsulation techniques for making vitamin premixes appear to be inadequate to guarantee VA, VK_3_ and VB_1_ concentrations in VTM premixes. Vitamin content can be used as a reliable index that reflects the history of vitamin and VTM premixes. Kinetic models of vitamin indices for vitamin and VTM premixes can be used to evaluate, control and properly manage the chain of premixes. We suggest that different options be available for the prediction of vitamin retention at different storage times. We also recommend reducing the time between vitamin premix production and feeding by farmers to minimize the loss of vitamins in diet formulations (especially vitamins A, K_3_ and B_1_). Additionally, for feed suppliers, we advise against the production of premixes that contain both trace minerals and vitamins.

## Figures and Tables

**Table 1:** Composition of the vitamin premixes during vitamin premix storage ^1^.

Item	Vitamin Premix 1	Vitamin Premix 2
Vitamin ^2^, unit/kg		
Vitamin A, IU	5,400,000	5,400,000
Vitamin D_3_, IU	1,200,000	1,200,000
Vitamin E, IU	12,000	12,000
Vitamin K_3_, mg	1200	1200
Vitamin B_1_, mg	1200	1200
Vitamin B_2_, mg	2400	2400
Vitamin B_6_, mg	1200	1200
Niacin, mg	12,000	12,000
Pantothenic acid, mg	7200	7200
Folic acid, mg	48	48
Biotin, mg	12	12
Vitamin B_12_, mg	9.6	9.6
Choline, mg	-	160,000

^1^ Vitamin premixes were designed to be added at a rate of 0.25% of the diet for weanling piglets. ^2^ Vitamin sources: Vitamin A; retinyl acetate, Vitamin D_3_; cholecalciferol, Vitamin E; D, L-α-tocopherol acetate, Vitamin K_3_; menadione sodium bisulfite, Vitamin B_1_; thiamine mononitrate, Vitamin B_2_; riboflavin, Vitamin B_6_; pyridoxine hydrochloride, Niacin; nicotinic acid, Pantothenic acid; D-calcium pantothenate, Vitamin B_7_; biotin, Vitamin B_9_; folic acid, Vitamin B_12_; cyanocobalamin and choline; choline chloride.

**Table 2:** Composition of the vitamin/trace mineral (VTM) premixes during storage ^1^.

Item	VTM Premix 1	VTM Premix 2	VTM Premix 3	VTM Premix 4
Vitamin ^2^, unit/kg				
Vitamin A, IU	1,350,000	1,350,000	1,350,000	1,350,000
Vitamin D_3_, IU	300,000	300,000	300,000	300,000
Vitamin E, IU	3000	3000	3000	3000
Vitamin K_3_, mg	300	300	300	300
Vitamin B_1_, mg	300	300	300	300
Vitamin B_2_, mg	600	600	600	600
Vitamin B_6_, mg	300	300	300	300
Niacin, mg	3000	3000	3000	3000
Pantothenic acid, mg	1800	1800	1800	1800
Folic acid, mg	12	12	12	12
Biotin, mg	3	3	3	3
Vitamin B_12_, mg	2.4	2.4	2.4	2.4
Choline, mg	-	40,000	-	40,000
Trace Mineral, mg/kg				
Cu (CuSO_4_)	500	500	20,000	20,000
I [Ca(IO_3_)_2_]	14	14	14	14
Fe (FeSO_4_)	10,000	10,000	10,000	10,000
Mn (MnO)	300	300	300	300
Se (NaSeO2)	25	25	25	25
Zn (ZnO)	8000	8000	225,000	225,000

^1^ The VTM premixes were designed to be added at a rate of 1% of the diet for weanling piglets. ^2^ Vitamin sources: Vitamin A; retinyl acetate, Vitamin D_3_; cholecalciferol, Vitamin E; D, L-α-tocopherol acetate, Vitamin K_3_; menadione sodium bisulfite, Vitamin B_1_; thiamine mononitrate, Vitamin B_2_; riboflavin, Vitamin B_6_; pyridoxine hydrochloride, Niacin; nicotinic acid, Pantothenic acid; D-calcium pantothenate, Vitamin B_7_; biotin, Vitamin B_9_; folic acid, Vitamin B_12_; cyanocobalamin and choline; choline chloride.

**Table 3:** Analyzed vitamin values in vitamin and vitamin/trace mineral (VTM) premixes ^1^.

Item	Vitamin Premix 1	Vitamin Premix 2	VTM Premix 1	VTM Premix 2	VTM Premix 3	VTM Premix 4
Vitamin ^2^, unit/kg						
Vitamin A, IU	5,423,890.96	5,423,890.96	1,313,201.26	1,331,795.34	1,336,480.19	1,342,884.50
Vitamin D_3_, IU	1,230,463.12	1,181,637.43	296,688.97	300,443.01	297,259.89	301,900.95
Vitamin E, IU	11,884.10	12,087.20	2938.45	2931.97	2978.32	3018.27
Vitamin K_3_, mg	1178.10	1183.14	305.85	295.95	298.61	302.94
Vitamin B_1_, mg	1165.00	1186.21	298.79	297.99	298.03	297.10
Vitamin B_2_, mg	2330.58	2348.85	596.55	589.76	590.07	606.20
Vitamin B_6_, mg	1184.88	1162.39	312.83	291.20	299.53	301.60
Niacin, mg	11,986.84	12,037.98	3007.11	2959.04	2979.74	2936.19
Pantothenic acid, mg	7166.46	7137.66	1840.95	1799.86	1836.04	1731.48

^1^ Vitamin premixes were designed to be added at a rate of 0.25% of the diet for weanling piglets, and VTM premixes were designed to be added at a rate of 1% of the diet for weanling piglets. ^2^ Values represent means of six replicate samples each analyzed in duplicate (method 2012.10 for VA and VE analysis; AOAC 2012, method 992.26 for VD_3_ analysis; AOAC 2012, GB/T 18872-2017 for VK_3_ analysis; National standard 2017, method for water soluble vitamins analysis; Chen et al. 2009, Ministry of Agriculture and Rural Affairs Feed Efficacy and Safety Evaluation Center, Beijing, CN).

**Table 4:** Retention (%) in vitamin A activity in different storage periods for vitamin and vitamin/trace mineral (VTM) premixes ^1^.

Items	Choline	Cu and Zn	Months (Time)					SEM	*p*-Time
			1	2	3	6	12		
Vitamin premix 1	-	-	99.61	98.98	99.30	98.96	97.75	1.04	0.767
Vitamin premix 2	+	-	99.77 ^a^	99.72 ^a^	99.10 ^a^	97.47 ^a^	90.19 ^b^	1.22	<0.01
		SEM	0.87	1.22	1.36	1.14	1.02		
	*p*-Choline		0.900	0.677	0.918	0.376	<0.01		
VTM premix 1	-	Low	99.21 ^a^	95.11 ^ABb^	93.39 ^Ab^	90.52 ^Ac^	86.96 ^Ad^	0.54	<0.01
VTM premix 2	+	Low	99.41 ^a^	96.02 ^Ab^	90.44 ^Bc^	85.80 ^Bd^	83.38 ^Be^	0.53	<0.01
VTM premix 3	-	High	99.72 ^a^	93.57 ^BCb^	89.27 ^Cc^	83.25 ^Cd^	77.02 ^Ce^	0.46	<0.01
VTM premix 4	+	High	99.30 ^a^	92.51 ^Cb^	85.74 ^Dc^	80.21 ^Dd^	74.42 ^De^	0.66	<0.01
		SEM	0.18	0.51	0.56	0.51	0.63		
	Main effects								
	Choline	-	99.46	94.34	91.33	86.89	81.99		
		+	99.35	94.26	88.09	83.00	78.90		
	Cu and Zn	Low	99.51	95.56	91.91	88.16	85.17		
		High	99.31	93.04	87.51	81.73	75.72		
	*p*-value								
	Choline		0.544	0.884	<0.01	<0.01	<0.01		
	Cu and Zn		0.271	<0.01	<0.01	<0.01	<0.01		
	Choline x Cu and Zn		0.099	0.067	0.606	0.115	0.446		

^1^ Vitamin premix 1 contained no choline chloride, while Vitamin premix 2 contained 160,000 mg/kg of choline chloride. VTM premix 1 contained 500 mg/kg of CuSO_4_ and 8000 mg/kg of ZnO, whereas VTM premix 2 contained 40,000 mg/kg of choline chloride, 500 mg/kg of CuSO_4_ and 8000 mg/kg of ZnO. VTM premix 3 contained 20,000 mg/kg of CuSO_4_ and 225,000 mg/kg of ZnO. VTM premix 4 contained 40,000 mg/kg of choline chloride, 20,000 mg/kg of CuSO_4_ and 225,000 mg/kg of ZnO. ^A, B, C, D^ Means in a column, with different superscripts, are different (*p* < 0.05). ^a, b, c, d, e^ Means in a row, with different superscripts, are different (*p* < 0.05).

**Table 5:** Retention (%) in vitamin D_3_ activity in different storage periods for vitamin and vitamin/trace mineral (VTM) premixes ^1^.

Items	Choline	Cu and Zn	Months (Time)					SEM	*p*-Time
			1	2	3	6	12		
Vitamin premix 1	-	-	96.67	94.65	91.20	91.30	93.14	1.50	0.077
Vitamin premix 2	+	-	97.80	96.31	93.55	95.57	94.45	1.25	0.150
		SEM	1.20	1.32	1.19	2.00	0.95		
	*p*-Choline		0.518	0.393	0.192	0.164	0.353		
VTM premix 1	-	Low	98.83 ^a^	97.33 ^a^	95.39 ^a^	91.60 ^b^	86.04 ^c^	0.86	<0.01
VTM premix 2	+	Low	99.15 ^a^	97.50 ^a^	96.43 ^a^	92.73 ^b^	85.24 ^c^	0.83	<0.01
VTM premix 3	-	High	98.79 ^a^	97.33 ^a^	95.17 ^ab^	91.53 ^b^	84.49 ^c^	1.08	<0.01
VTM premix 4	+	High	98.83 ^a^	95.76 ^a^	96.14 ^a^	89.83 ^b^	84.87 ^c^	1.04	<0.01
		SEM	0.47	1.21	1.15	1.03	0.70		
	Main effects								
	Choline	-	98.81	97.33	95.28	91.57	85.27		
		+	98.99	96.30	96.29	91.28	85.06		
	Cu and Zn	Low	98.98	97.41	95.91	92.16	85.64		
		High	98.81	96.23	95.66	90.68	84.68		
	*p*-value								
	Choline		0.713	0.410	0.394	0.784	0.768		
	Cu and Zn		0.710	0.339	0.826	0.166	0.191		
	Choline x Cu and Zn		0.762	0.338	0.974	0.186	0.412		

^1^ Vitamin premix 1 contained no choline chloride, while Vitamin premix 2 contained 160,000 mg/kg of choline chloride. VTM premix 1 contained 500 mg/kg of CuSO_4_ and 8000 mg/kg of ZnO, and VTM premix 2 contained 40,000 mg/kg of choline chloride, 500 mg/kg of CuSO_4_ and 8000 mg/kg of ZnO. VTM premix 3 contained 20,000 mg/kg of CuSO_4_ and 225,000 mg/kg of ZnO. VTM premix 4 contained 40,000 mg/kg of choline chloride, 20,000 mg/kg of CuSO_4_ and 225,000 mg/kg of ZnO. ^a, b, c^ Means in a row, with different superscripts, are different (*p* < 0.05).

**Table 6:** Retention (%) in vitamin E activity in different storage periods for vitamin and vitamin/trace mineral (VTM) premixes ^1^.

Items	Choline	Cu and Zn	Months (Time)					SEM	*p*-Time
			1	2	3	6	12		
Vitamin premix 1	-	-	99.01	97.57	96.98	95.49	95.96	1.76	0.685
Vitamin premix 2	+	-	97.38	97.24	96.77	99.48	95.70	1.08	0.185
		SEM	0.87	1.17	0.55	2.68	1.06		
	*p*-Choline		0.197	0.851	0.797	0.318	0.870		
VTM premix 1	-	Low	98.70 ^a^	97.04 ^ab^	96.94 ^ab^	97.74 ^ab^	93.93 ^b^	0.97	<0.01
VTM premix 2	+	Low	99.61 ^a^	96.57 ^ab^	96.70 ^ab^	97.44 ^ab^	93.32 ^b^	1.25	0.028
VTM premix 3	-	High	99.18 ^a^	96.99 ^ab^	96.60 ^ab^	96.52 ^ab^	94.08 ^b^	0.76	<0.01
VTM premix 4	+	High	99.29 ^a^	98.20 ^ab^	96.38 ^bc^	97.55 ^ab^	93.82 ^c^	0.63	<0.01
		SEM	0.32	0.65	1.02	1.63	0.57		
	Main effects								
	Choline	-	98.94	97.01	96.29	97.13	94.00		
		+	99.45	97.38	96.54	97.50	93.57		
	Cu and Zn	Low	99.16	96.80	96.34	97.59	93.63		
		High	99.23	97.59	96.49	97.03	93.95		
	*p*-value								
	Choline		0.130	0.582	0.748	0.835	0.455		
	Cu and Zn		0.822	0.241	0.851	0.750	0.577		
	Choline x Cu and Zn		0.229	0.214	0.556	0.705	0.760		

^1^ Vitamin premix 1 contained no choline chloride, while Vitamin premix 2 contained 160,000 mg/kg of choline chloride. VTM premix 1 contained 500 mg/kg of CuSO_4_ and 8000 mg/kg of ZnO, and in addition, VTM premix 2 contained 40,000 mg/kg of choline chloride, 500 mg/kg of CuSO_4_ and 8000 mg/kg of ZnO. VTM premix 3 contained 20,000 mg/kg of CuSO_4_ and 225,000 mg/kg of ZnO. VTM premix 4 contained 40,000 mg/kg of choline chloride, 20,000 mg/kg of CuSO_4_ and 225,000 mg/kg of ZnO. ^a, b, c^ Means in a row, with different superscripts, are different (*p* < 0.05).

**Table 7:** Retention (%) in vitamin B_6_ activity in different storage periods for vitamin and vitamin/trace mineral (VTM) premixes ^1^.

Items	Choline	Cu and Zn	Months (Time)					SEM	*p*-Time
			1	2	3	6	12		
Vitamin premix 1	-	-	98.66	97.82	96.79	95.78	95.33	1.17	0.221
Vitamin premix 2	+	-	97.26 ^a^	96.73 ^a^	95.40 ^a^	97.30 ^a^	92.17 ^b^	0.69	<0.01
		SEM	0.88	0.56	0.86	0.99	1.53		
	*p*-Choline		0.288	0.176	0.057	0.304	0.018		
VTM premix 1	-	Low	99.01 ^a^	98.00 ^ab^	97.13 ^ab^	93.68 ^bc^	90.86 ^c^	1.18	<0.01
VTM premix 2	+	Low	98.96 ^a^	97.21 ^ab^	97.77 ^a^	94.96 ^a^	90.26 ^b^	1.06	<0.01
VTM premix 3	-	High	98.86 ^a^	98.36 ^a^	96.79 ^ab^	93.69 ^ab^	91.01 ^b^	1.47	<0.01
VTM premix 4	+	High	98.84 ^a^	98.24 ^a^	97.82 ^ab^	93.31 ^bc^	91.38 ^c^	1.15	<0.01
		SEM	0.36	1.67	1.80	1.08	0.43		
	Main effects								
	Choline	-	98.93	98.18	96.96	93.68	90.94		
		+	98.90	97.72	97.79	94.14	90.82		
	Cu and Zn	Low	98.98	97.60	97.45	94.32	90.56		
		High	98.85	98.30	97.31	93.50	91.19		
	*p*-value								
	Choline		0.928	0.787	0.649	0.679	0.789		
	Cu and Zn		0.714	0.682	0.938	0.458	0.157		
	Choline x Cu and Zn		0.971	0.844	0.916	0.451	0.273		

^1^ Vitamin premix 1 contained no choline chloride, whereas Vitamin premix 2 contained 160,000 mg/kg of choline chloride. VTM premix 1 contained 500 mg/kg of CuSO_4_ and 8000 mg/kg of ZnO, and VTM premix 2 contained 40,000 mg/kg of choline chloride, 500 mg/kg of CuSO_4_ and 8000 mg/kg of ZnO. VTM premix 3 contained 20,000 mg/kg of CuSO_4_ and 225,000 mg/kg of ZnO. VTM premix 4 contained 40,000 mg/kg of choline chloride, 20,000 mg/kg of CuSO_4_ and 225,000 mg/kg of ZnO. ^a, b, c^ Means in a row, with different superscripts, are different (*p* < 0.05).

**Table 8:** Retention (%) in niacin activity in different storage periods for vitamin and vitamin/trace mineral (VTM) premixes ^1^.

Items	Choline	Cu and Zn	Months (Time)					SEM	*p*-Time
			1	2	3	6	12		
Vitamin premix 1	-	-	99.67	97.89	99.76	98.90	98.54	0.56	0.130
Vitamin premix 2	+	-	98.60	99.35	98.87	98.54	98.43	0.90	0.954
		SEM	1.18	0.89	0.37	0.66	0.34		
	*p*-Choline		0.557	0.501	0.843	0.149	0.472		
VTM premix 1	-	Low	99.58 ^a^	97.46 ^ab^	97.05 ^ab^	95.43 ^bc^	93.66 ^c^	0.69	<0.01
VTM premix 2	+	Low	99.08 ^a^	98.14 ^a^	97.91 ^a^	95.28 ^b^	93.28 ^b^	0.89	<0.01
VTM premix 3	-	High	99.38 ^a^	97.33 ^a^	97.78 ^a^	94.89 ^b^	93.94 ^b^	0.55	<0.01
VTM premix 4	+	High	98.36 ^a^	97.37 ^ab^	96.77 ^abc^	94.53 ^bc^	93.32 ^c^	0.93	<0.01
		SEM	0.38	1.07	0.94	1.39	0.49		
	Main effects								
	Choline	-	99.48	97.39	97.41	95.16	93.80		
		+	98.72	97.76	97.34	94.91	93.30		
	Cu and Zn	Low	99.33	97.80	97.48	95.36	93.47		
		High	98.87	97.35	97.27	94.71	93.63		
	*p*-value								
	Choline		0.059	0.737	0.940	0.859	0.314		
	Cu and Zn		0.239	0.679	0.827	0.648	0.745		
	Choline x Cu and Zn		0.492	0.767	0.331	0.943	0.807		

^1^ Vitamin premix 1 contained no choline chloride, and yet Vitamin premix 2 contained 160,000 mg/kg of choline chloride. VTM premix 1 contained 500 mg/kg of CuSO_4_ and 8000 mg/kg of ZnO, while VTM premix 2 contained 40,000 mg/kg of choline chloride, 500 mg/kg of CuSO_4_ and 8000 mg/kg of ZnO. VTM premix 3 contained 20,000 mg/kg of CuSO_4_ and 225,000 mg/kg of ZnO. VTM premix 4 contained 40,000 mg/kg of choline chloride, 20,000 mg/kg of CuSO_4_ and 225,000 mg/kg of ZnO. ^a, b, c^ Means in a row, with different superscripts, are different (*p* < 0.05).

**Table 9:** Retention (%) in pantothenic acid activity in different storage periods for vitamin and vitamin/trace mineral (VTM) premixes ^1^.

Items	Choline	Cu and Zn	Months (Time)					SEM	*p*-Time
			1	2	3	6	12		
Vitamin premix 1	-	-	99.25	96.30	96.05	96.60	96.97	0.83	0.054
Vitamin premix 2	+	-	98.10	95.22	95.17	95.14	96.54	1.68	0.671
		SEM	0.44	0.90	1.09	1.36	1.90		
	*p*-Choline		0.079	0.413	0.481	0.135	0.877		
VTM premix 1	-	Low	99.58 ^a^	98.22 ^a^	96.84 ^ab^	95.62 ^ab^	93.79 ^b^	1.03	<0.01
VTM premix 2	+	Low	99.41 ^a^	98.32 ^a^	96.38 ^ab^	97.69 ^ab^	93.63 ^b^	1.10	0.013
VTM premix 3	-	High	99.72 ^a^	98.68 ^a^	96.48 ^ab^	96.02 ^ab^	93.17 ^b^	1.12	<0.01
VTM premix 4	+	High	99.72 ^a^	98.38 ^ab^	96.57 ^ab^	96.71 ^ab^	93.83 ^b^	0.99	0.029
		SEM	0.91	0.78	1.54	1.11	0.83		
	Main effects								
	Choline	-	99.65	98.45	96.66	95.81	93.48		
		+	99.06	98.36	96.48	97.20	93.73		
	Cu and Zn	Low	99.49	98.28	96.61	96.65	93.71		
		High	99.22	98.54	96.53	96.36	93.50		
	*p*-value								
	Choline		0.528	0.902	0.907	0.270	0.768		
	Cu and Zn		0.766	0.744	0.955	0.815	0.806		
	Choline x Cu and Zn		0.654	0.807	0.860	0.576	0.627		

^1^ Vitamin premix 1 contained no choline chloride, and Vitamin premix 2 contained 160,000 mg/kg of choline chloride. VTM premix 1 contained 500 mg/kg of CuSO_4_ and 8000 mg/kg of ZnO, while VTM premix 2 contained 40,000 mg/kg of choline chloride, 500 mg/kg of CuSO_4_ and 8000 mg/kg of ZnO. VTM premix 3 contained 20,000 mg/kg of CuSO_4_ and 225,000 mg/kg of ZnO. VTM premix 4 contained 40,000 mg/kg of choline chloride, 20,000 mg/kg of CuSO_4_ and 225,000 mg/kg of ZnO. ^a, b^ Means in a row, with different superscripts, are different (*p* < 0.05).

**Table 10:** Retention (%) in vitamin K_3_ activity in different storage periods for vitamin and vitamin/trace mineral (VTM) premixes ^1^.

Items	Choline	Cu and Zn	Months (Time)					SEM	*p*-Time
			1	2	3	6	12		
Vitamin premix 1	-	-	97.43 ^a^	94.32 ^a^	85.76 ^b^	79.09 ^c^	64.62 ^d^	1.60	<0.01
Vitamin premix 2	+	-	96.68 ^a^	94.05 ^a^	85.03 ^b^	74.61 ^c^	60.06 ^d^	1.78	<0.01
		SEM	0.78	0.77	1.17	1.85	2.89		
	*p*-Choline		0.513	0.807	0.669	0.119	0.291		
VTM premix 1	-	Low	98.32 ^a^	93.26 ^b^	92.51 ^b^	70.80 ^c^	53.39 ^d^	0.62	<0.01
VTM premix 2	+	Low	96.96 ^a^	93.14 ^b^	91.25 ^b^	70.61 ^c^	52.29 ^d^	0.75	<0.01
VTM premix 3	-	High	98.27 ^a^	93.35 ^b^	92.37 ^b^	69.95 ^c^	53.35 ^d^	0.73	<0.01
VTM premix 4	+	High	98.87 ^a^	92.95 ^b^	92.60 ^b^	71.34 ^c^	52.79 ^d^	0.79	<0.01
		SEM	0.74	0.55	0.79	0.80	0.71		
	Main effects								
	Choline	-	98.30	93.31	92.44	70.38	53.37		
		+	97.91	93.04	91.92	70.97	52.54		
	Cu and Zn	Low	97.64	93.20	91.88	70.71	52.84		
		High	98.57	93.15	92.48	70.64	53.07		
	*p*-value								
	Choline		0.610	0.639	0.517	0.463	0.257		
	Cu and Zn		0.221	0.931	0.452	0.936	0.753		
	Choline x Cu and Zn		0.199	0.807	0.358	0.335	0.708		

^1^ Vitamin premix 1 contained no choline chloride, Vitamin premix 2 contained 160,000 mg/kg of choline chloride. VTM premix 1 contained 500 mg/kg of CuSO_4_ and 8000 mg/kg of ZnO, and VTM premix 2 contained 40,000 mg/kg of choline chloride, 500 mg/kg of CuSO_4_ and 8000 mg/kg of ZnO. VTM premix 3 contained 20,000 mg/kg of CuSO_4_ and 225,000 mg/kg of ZnO. VTM premix 4 contained 40,000 mg/kg of choline chloride, 20,000 mg/kg of CuSO_4_ and 225,000 mg/kg of ZnO. ^a, b, c, d^ Means in a row, with different superscripts, are different (*p* < 0.05).

**Table 11:** Retention (%) in vitamin B_1_ activity in different storage periods for vitamin and vitamin/trace mineral (VTM) premixes ^1^.

Items	Choline	Cu and Zn	Months (Time)					SEM	*p*-Time
			1	2	3	6	12		
Vitamin premix 1	-	-	97.82 ^a^	98.34 ^a^	97.02 ^a^	94.07 ^ab^	91.18 ^b^	1.11	<0.01
Vitamin premix 2	+	-	98.28 ^a^	99.02 ^a^	98.30 ^a^	97.07 ^a^	91.90 ^b^	0.97	<0.01
		SEM	0.62	0.80	0.68	1.80	0.84		
	*p*-Choline		0.612	0.549	0.211	0.269	0.560		
VTM premix 1	-	Low	98.49 ^a^	96.62 ^Aa^	91.53 ^Ab^	91.74 ^Ab^	88.55 ^Ac^	0.66	<0.01
VTM premix 2	+	Low	99.46 ^a^	85.57 ^Bb^	83.45 ^Bb^	76.14 ^Bc^	70.98 ^Bd^	1.02	<0.01
VTM premix 3	-	High	99.07 ^a^	81.07 ^Cb^	63.30 ^Cc^	49.63 ^Cd^	34.80 ^Ce^	0.79	<0.01
VTM premix 4	+	High	99.24 ^a^	79.91 ^Cb^	60.20 ^Cc^	46.80 ^Dd^	34.11 ^Ce^	0.86	<0.01
		SEM	0.39	0.48	1.19	0.59	1.18		
	Main effects								
	Choline	-	98.78	88.84	77.41	70.68	61.67		
		+	99.35	82.74	71.82	61.47	52.55		
	Cu and Zn	Low	98.98	91.09	87.49	83.94	79.77		
		High	99.15	80.49	61.75	48.21	34.45		
	*p*-value								
	Choline		0.164	<0.01	<0.01	<0.01	<0.01		
	Cu and Zn		0.661	<0.01	<0.01	<0.01	<0.01		
	Choline x Cu and Zn		0.327	<0.01	0.05	<0.01	<0.01		

^1^ Vitamin premix 1 contained no choline chloride, while in addition, Vitamin premix 2 contained 160,000 mg/kg of choline chloride. VTM premix 1 contained 500 mg/kg of CuSO_4_ and 8000 mg/kg of ZnO, and VTM premix 2 contained 40,000 mg/kg of choline chloride, 500 mg/kg of CuSO_4_ and 8000 mg/kg of ZnO. VTM premix 3 contained 20,000 mg/kg of CuSO_4_ and 225,000 mg/kg of ZnO. VTM premix 4 contained 40,000 mg/kg of choline chloride, 20,000 mg/kg of CuSO_4_ and 225,000 mg/kg of ZnO. ^A, B, C, D^ Means in a column, with different superscripts, are different (*p* < 0.05). ^a, b, c, d, e^ Means in a row, with different superscripts, are different (*p* < 0.05).

**Table 12:** Retention (%) in vitamin B_2_ activity in different storage periods for vitamin and vitamin/trace mineral (VTM) premixes ^1^.

Items	Choline	Cu and Zn	Months (Time)					SEM	*p*-Time
			1	2	3	6	12		
Vitamin premix 1	-	-	99.05 ^a^	96.29 ^ab^	95.08 ^ab^	95.16 ^ab^	91.40 ^b^	1.27	<0.01
Vitamin premix 2	+	-	98.69 ^a^	94.76 ^ab^	93.89 ^ab^	93.52 ^b^	94.52 ^b^	0.96	0.012
		SEM	0.55	0.78	1.11	1.54	1.33		
	*p*-Choline		0.635	0.195	0.464	0.470	0.128		
VTM premix 1	-	Low	99.41 ^a^	97.95 ^ab^	96.08 ^ab^	94.22 ^ab^	92.14 ^b^	1.43	0.011
VTM premix 2	+	Low	98.67 ^a^	98.09 ^ab^	96.42 ^ab^	98.22 ^a^	94.05 ^b^	1.12	0.02
VTM premix 3	-	High	98.51 ^a^	98.71 ^a^	96.01 ^ab^	94.27 ^b^	92.82 ^b^	0.97	<0.01
VTM premix 4	+	High	99.04 ^a^	98.48 ^a^	96.03 ^ab^	94.78 ^b^	92.86 ^b^	0.89	<0.01
		SEM	0.55	0.50	0.35	2.26	0.70		
	Main effects								
	Choline	-	98.96	98.33	96.04	94.24	92.48		
		+	98.86	98.29	96.22	96.50	93.45		
	Cu and Zn	Low	99.04	98.02	96.25	96.22	93.10		
		High	98.78	98.60	96.02	94.52	92.84		
	*p*-value								
	Choline		0.857	0.938	0.614	0.330	0.181		
	Cu and Zn		0.639	0.265	0.511	0.463	0.716		
	Choline x Cu and Zn		0.264	0.713	0.651	0.450	0.199		

^1^ Vitamin premix 1 contained no choline chloride, while Vitamin premix 2 contained 160,000 mg/kg of choline chloride. VTM premix 1 contained 500 mg/kg of CuSO_4_ and 8000 mg/kg of ZnO, and then VTM premix 2 contained 40,000 mg/kg of choline chloride, 500 mg/kg of CuSO_4_ and 8000 mg/kg of ZnO. VTM premix 3 contained 20,000 mg/kg of CuSO_4_ and 225,000 mg/kg of ZnO. VTM premix 4 contained 40,000 mg/kg of choline chloride, 20,000 mg/kg of CuSO_4_ and 225,000 mg/kg of ZnO. ^a, b^ Means in a row, with different superscripts, are different (*p* < 0.05).

**Table 13:** Prediction equations for vitamin retention (%) in different vitamin/trace mineral (VTM) premixes during storage.

No.	Types ^1^	Vitamin Sources	Predicted Equations ∗	*R* ^2^	RMSEP	*p*-Value
1	VTM premix 1	VA	y = 97.777e^−0.011x^	0.884	0.02	0.017
2	VTM premix 1	VD_3_	y = −1.147x + 99.343	0.984	0.752	0.001
3	VTM premix 1	VE	y = −0.351x + 98.556	0.761	1.01	0.054
4	VTM premix 1	VK_3_	y = −4.212x + 101.873	0.966	4.045	0.003
5	VTM premix 1	VB_1_	y = −0.786x + 97.157	0.736	2.41	0.063
6	VTM premix 1	VB_2_	y = −0.651x + 98.914	0.891	1.104	0.016
7	VTM premix 1	VB_6_	y = −0.747x + 99.322	0.96	0.779	0.003
8	VTM premix 1	Niacin	y = 98.913e^−0.005x^	0.888	0.009	0.016
9	VTM premix 1	Pantothenic acid	y = −0.478x + 99.106	0.893	0.849	0.015
10	VTM premix 2	VA	y = −1.347x + 97.474	0.788	3.581	0.044
11	VTM premix 2	VD_3_	y = −1.248x + 100.2	0.999	0.165	<0.001
12	VTM premix 2	VE	y = −0.433x + 98.808	0.723	1.374	0.068
13	VTM premix 2	VK_3_	y = −4.217x + 101.09	0.973	3.569	0.002
14	VTM premix 2	VB_1_	y = 93.34e^−0.026x^	0.785	0.068	0.045
15	VTM premix 2	VB_2_	y = 98.821e^−0.004x^	0.697	0.013	0.078
16	VTM premix 2	VB_6_	y = −0.766x + 99.511	0.98	0.564	0.001
17	VTM premix 2	Niacin	y = −0.528x + 99.271	0.958	0.567	0.004
18	VTM premix 2	Pantothenic acid	y = 99.252e^−0.005x^	0.799	0.012	0.041
19	VTM premix 3	VA	y = −1.868x + 97.531	0.882	3.495	0.018
20	VTM premix 3	VD_3_	y = −1.283x + 99.618	0.993	0.538	<0.001
21	VTM premix 3	VE	y = −0.37x + 98.449	0.82	0.888	0.034
22	VTM premix 3	VK_3_	y = −4.226x + 101.743	0.96	4.409	0.003
23	VTM premix 3	VB_1_	y = 94.133e^−0.089x^	0.92	0.134	0.01
24	VTM premix 3	VB_2_	y = −0.534x + 98.629	0.842	1.186	0.028
25	VTM premix 3	VB_6_	y = 99.305e^−0.008^	0.955	0.009	0.004
26	VTM premix 3	Niacin	y = −0.456x + 98.853	0.835	1.04	0.03
27	VTM premix 3	Pantothenic acid	y = 99.451e^−0.006x^	0.904	0.009	0.013
28	VTM premix 4	VA	y = −2.013x + 96.098	0.827	4.715	0.032
29	VTM premix 4	VD_3_	y = −1.242x + 99.048	0.953	1.415	0.004
30	VTM premix 4	VE	y = −0.421x + 99.069	0.8	1.08	0.041
31	VTM premix 4	VK_3_	y = −4.276x + 102.235	0.971	3.764	0.002
32	VTM premix 4	VB_1_	y = 92.009e^−0.09x^	0.89	0.162	0.016
33	VTM premix 4	VB_2_	y = −0.541x + 98.834	0.871	1.065	0.02
34	VTM premix 4	VB_6_	y = −0.721x + 99.38	0.912	1.148	0.011
35	VTM premix 4	Niacin	y = −0.447x + 98.217	0.908	0.728	0.012
36	VTM premix 4	Pantothenic acid	y = 99.29e^−0.005x^	0.872	0.009	0.02

^1^ RMSEP, root mean square error of prediction. VTM premix 1 contained low concentrations of Cu and Zn without choline. VTM premix 2 contained choline with low concentrations of Cu and Zn. VTM premix 3 contained high concentrations of Cu and Zn without choline. VTM premix 4 contained choline with high concentrations of Cu and Zn. ∗ y (%) is retention of vitamin, x (month) is storage time.
